# Differential Response to Trichloroethylene-Induced Hepatosteatosis in Wild-Type and PPARα-Humanized Mice

**DOI:** 10.1289/ehp.1001928

**Published:** 2010-08-13

**Authors:** Doni Hikmat Ramdhan, Michihiro Kamijima, Dong Wang, Yuki Ito, Hisao Naito, Yukie Yanagiba, Yumi Hayashi, Naoki Tanaka, Toshifumi Aoyama, Frank J. Gonzalez, Tamie Nakajima

**Affiliations:** 1 Department of Occupational and Environmental Health, Nagoya University Graduate School of Medicine, Nagoya, Japan; 2 Department of Occupational and Environmental Health, Nagoya City University Graduate School of Medical Sciences, Nagoya, Japan; 3 Department of Metabolic Regulation, Shinshu University Graduate School of Medicine, Matsumoto, Japan; 4 Laboratory of Metabolism, National Cancer Institute, National Institutes of Health, Department of Health and Human Services, Bethesda, Maryland, USA

**Keywords:** CYP2E1, fatty acid β-oxidation, hepatotoxicity, PPARα, steatosis, trichloroethylene

## Abstract

**Background:**

Trichloroacetic acid, an oxidative metabolite of trichloroethylene (TRI), is a ligand of the peroxisome proliferator-activated receptor α (PPAR) α, which is involved in lipid homeostasis and anti-inflammation.

**Objective:**

We examined the role of mouse and human PPARα in TRI-induced hepatic steatosis and toxicity.

**Methods:**

Male wild-type (m*PPAR*α), *Ppar*α-null, and humanized PPARα (h*PPAR*α) mice on an Sv/129 background were exposed via inhalation to 0, 1,000, and 2,000 ppm TRI for 8 hr/day for 7 days. We assessed TRI-induced steatosis or hepatic damage through biochemical and histopathological measurements.

**Results:**

Plasma alanine aminotransferase and aspartate aminotransferase activities increased in all mouse lines after exposure to 1,000 and 2,000 ppm TRI. Exposure induced hepatocyte necrosis and inflammatory cells in all mouse lines, but hepatic lipid accumulation was observed only in *Ppar*α-null and h*PPAR*α mice. No differences were observed in TRI-mediated induction of hepatic PPARα target genes except for a few genes that differed between m*PPAR*α and h*PPAR*α mice. However, TRI significantly increased expression of triglyceride (TG)-synthesizing enzymes, diacylglicerol acyltransferases, and PPARγ in *Ppar*α-null and h*PPAR*α mice, which may account for the increased TG in their livers. TRI exposure elevated nuclear factor-kappa B (NFκB) *p52* mRNA and protein in all mice regardless of *PPAR*α genotype.

**Conclusions:**

NFκB-p52 is a candidate molecular marker for inflammation caused by TRI, and PPARα may be involved in TRI-induced hepatosteatosis. However, human PPARα may afford only weak protection against TRI-mediated effects compared with mouse PPARα.

Trichloroethylene (TRI) is a chlorinated solvent that has been used as a degreaser and as an intermediate in synthesizing other chemicals. Occupational TRI exposure is declining in industrialized countries because of technological innovation and legislation (Grote et al. 2003), but it is increasing in emerging industrialized countries, especially in Asia (Huang et al. 2002). TRI exposure has been associated with rat renal (Mensing et al. 2002) and mouse liver injury (Ramdhan et al. 2008), impaired reproductive function in male mice (Forkert et al. 2002), autoimmune hepatitis in the autoimmune-prone MRL mouse model (Griffin et al. 2000), and allergic hepatitis in the guinea pig maximization test (Tang et al. 2008). Recently, occupational exposure to TRI was associated with severe hypersensitive skin damage and impaired hepatic function (Kamijima et al. 2007).

TRI is metabolized through oxidation by cytochrome P450 (CYP) isozymes such as CYP1A1/2, CYP2B1/2, CYP2C11, and CYP2E1 (Kim and Ghanayem 2006; Lash et al. 2000; Nakajima et al. 1990). CYP2E1 is the most important enzyme in the conversion of TRI to chloral hydrate through intermediate metabolite(s) formation (Nakajima et al. 1992; Ramdhan et al. 2008), which is rapidly metabolized to trichloroacetic acid (TCA) by aldehyde dehydrogenase (ALDH) or to trichloroethanol (TCE) by alcohol dehydrogenase (ADH). TCE can be converted to TCA by ADH and ALDH via chloral hydrate. In addition, a small portion of TRI is conjugated by glutathione *S*-transferase (GST) to form *S*-(dichlorovinyl) glutathione and further metabolized to *S*-(1,2-dichlorovinyl)-l cysteine, which is considered a kidney carcinogen (Lash et al. 2000). An intermediate metabolite(s) of TRI to chloral hydrate is thought to induce hepatic damage (Nakajima et al. 1988).

Peroxisome proliferators function as ligands for the peroxisome proliferator-activated receptor α (PPARα) (Issemann and Green 1990), a nuclear receptor that regulates genes involved in fatty acid transport and β-oxidation, resulting in increased fatty acid catabolism and increases in the number and size of peroxisomes in the livers of exposed mice or rats (Reddy and Krishnakantha 1975). TCA and DCA both activate PPARα in the liver, but other TRI metabolites do not (Maloney and Waxman 1999). Laughter et al. (2004) also reported the activation of PPARα by TRI, TCA, or DCA treatment in PPARα wild-type mice, judging from the induction of PPARα-related gene expressions, but not in *Ppar*α-null mice. In addition, Ramdhan et al. (2008) reported that PPARα was activated after TRI exposure in wild-type but not *CYP2E1*-null mice, which they attributed to a lack of CYP2E1-mediated production of TCA.

Whether activation of PPARα is involved in TRI-induced liver injury remains to be clarified. PPARα activation is accompanied by increased hepatic lipid turnover via the β-oxidation system, which reduces lipid accumulation that can result in hepatic toxicity (Harano et al. 2006). PPARα activation also can inhibit proinflammatory nuclear factor-kappa B (NFκB) by interacting with the p65 and p50 heterodimer (Delerive et al. 1999), and anti-inflammatory effects via PPARα-mediated inhibition of NFκB DNA binding activity have been demonstrated in human hepatocytes (Hirano et al. 2002). However, although the consequences of PPARα activation in the mouse liver is relatively well defined (Mandard et al. 2004), the consequences of human PPARα activation in response to TRI and other peroxisome proliferators is still poorly understood.

Another PPAR subtype, PPARγ, regulates genes involved in adipogenesis and lipid metabolism (Berger and Moller 2002). PPARγ may also be involved in TRI-induced hepatosteatosis, as TCA also activates mouse PPARγ (Maloney and Waxman 1999), and guinea pigs suffering from TRI-induced toxic liver injury showed fatty changes of the liver (Tang et al. 2008). In the present study, we investigated TRI-induced subacute hepatotoxicity in m*PPAR*α, *Ppar*α-null and humanized PPARα (h*PPAR*α) mice to clarify differences in the roles of human and mouse PPARα in TRI toxicity.

## Materials and Methods

### Chemicals

TRI was provided by the Japan Association for Hygiene of Chlorinated Solvents, and its purity was determined to be at least 99% as judged by gas chromatography-mass spectrometry (GC-MS). All other chemicals were purchased from commercial sources and were of the highest purity available.

### Animals

This study was conducted according to the Animal Experimental Guidelines of the Nagoya University Graduate School of Medicine. Male wild-type (m*PPAR*α*)*, *Ppar*α-null (Lee et al. 1995), and h*PPAR*α^Tet-OFF^ (h*PPAR*α) (Cheung et al. 2004) mice on an Sv/129 genetic background were bred as described elsewhere (Nakamura et al. 2009) and reared at the Institute of Laboratory Animal Research (Nagoya University Graduate School of Medicine). The expression of human PPARα cDNA in h*PPAR*α^Tet-OFF^ mice is limited to hepatocytes and is under the control of the tetracycline regulatory system, as described by Cheung et al. (2004). All mice were housed in a room under a 12:12 hr light:dark cycle (lights on at 0900 hours and off at 2100 hours), with stable relative humidity (57–60%) and a constant temperature (23–25°C). Food and water were provided *ad libitum*, and all animals were treated humanely and with regard for the alleviation of suffering. When the mice were 10 weeks of age, each strain was randomly divided into three groups of six animals each.

### TRI exposure

Each group of mice was exposed to 0, 1,000, or 2,000 ppm TRI [equivalent to 0, 800, and 1,600 mg/kg/day by gavage, respectively (Griffin et al. 2000)] or fresh air in an inhalation chamber for 8 hr/day over 7 consecutive days, as described previously (Ramdhan et al. 2008). In PPARα wild-type mice, TRI metabolism was previously shown to be saturated with exposure to approximately 1,000 ppm (Ramdhan et al. 2008), but we evaluated exposure to 1,000 and 2,000 ppm because of limited information concerning TRI metabolism in *Ppar*α-null and h*PPAR*α mice. We studied exposure by inhalation, because this is the most common route of occupational exposure in humans. Occupational exposures are generally much lower than exposures used in this research but may approach these levels in some cases, for example, during defatting processes using immersion tanks (Nakajima et al. 1980). Mice were moved to individual metal metabolism cages to collect urine samples after 7 days, and were sacrificed by exsanguination through the abdominal aorta under pentobarbital anesthesia the following day. Plasma was separated from whole blood by centrifugation at 3,000 rpm for 10 min. The liver was carefully dissected out and immediately weighed, and a small section was excised from the median lobe of each mouse and fixed in 10% neutral buffered formalin.

### Plasma aminotransferase activities

We measured aspartate aminotransferase (AST) and alanine aminotransferase (ALT) activities by the colorimetric method using a Transaminase C II Test Kit (Wako, Osaka, Japan).

### Triglyceride concentration

Lipid was extracted from livers using the method of Folch et al. (1957), and hepatic and plasma triglyceride (TG) were measured by the colorimetric method using TG-IE kits (Wako).

### Urinary metabolites

We measured urinary TCA and TCE concentrations by gas chromatography-mass spectrometry (GC-MS) (6890N gas chromatograph, 5975 Mass Selective Detector, 7683 Automatic Liquid Sampler; Agilent Technologies, Santa Clara, CA, USA), as described previously (Ramdhan et al. 2008).

### Histopathology evaluation

Tissue blocks were embedded in paraffin and 5-μm sections were mounted on glass slides and stained with hematoxylin and eosin (H&E). Photomicrographic images were captured on a BZ-8000 fluorescence microscope microscope (Keyence, Osaka, Japan). We identified steatosis based on the presence of vacuoles consistent with lipid accumulation (Kumar et al. 2007); steatosis was classified as macrovesicular steatosis if the nucleus was displaced by the vacuole or as microvesicular steatosis if the nucleus remained in the center of the hepatocyte (Brunt et al. 1999). Hepatocyte proliferation was classified based on the presence of enlarged hepatocytes with prominent eosinophilic cytoplasm (Yang et al. 2007). Cells with eosinophilic cytoplasm and pycnotic or karyolitic nuclei were designated as necrotic.

Histopathological findings in 20 randomly selected 200× microscopic fields per section were scored (Brunt et al. 1999; Okiyama et al. 2009) for steatosis [0, none; 1, mild (5–33% of parenchymal involvement of steatosis); 2, moderate (33–66%); or 3, severe (> 66%)]; necrotic cells [0, no necrosis; 1, minimal (only occasional necrotic cells in any lobule); 2, mild (less than one-third of the lobular structure affected); 3, moderate (one-third to two-thirds of lobular structure affected); or 4, severe (greater than two-thirds of the lobular structure affected)]; lobular and portal tract inflammation [0, none; 1, mild (< 2 foci/field); 2, moderate (2–4 foci/field); or 3, severe (> 4 foci/field)]; and hepatocyte proliferation (0, absent; 1, present).

### Real-time quantitative polymerase chain reaction (PCR)

We isolated total RNA from whole liver using the RNeasy Mini Kit (QIAGEN, Tokyo, Japan). Real-time PCR analysis was performed as described elsewhere (Ito et al. 2007; Nakamura et al. 2009; Ramdhan et al. 2008).

### Western blot analysis

A section of liver from each mouse was homogenized with three volumes of 10 mM phosphate buffer (pH 7.4) containing 0.25 M sucrose. The nuclear fraction (derived from both hepatocytes and nonparenchymal cells) was extracted using a CelLytic NuCLEAR Extraction Kit (Sigma, Tokyo, Japan). Nuclear fractions (NFκB-p65, NFκB-p50, NFκB-p52, and PPARα) and liver homogenates (other proteins include CYP2E1 and ALDH2) were subjected to 10% or 12.5% polyacrylamide gel electrophoresis, as described elsewhere (Aoyama et al. 1998; Kamijo et al. 2007; Ramdhan et al. 2008).

### Statistical analysis

Data are expressed as mean ± SD. We used the Tukey-Kramer HSD test to compare genotype effects (vs. wild-type controls) and exposure effects in each strain. TCA and TCE were below the detection limit in all control groups, and metabolism was saturated at 1,000 ppm. Therefore, we compared levels of TCA and TCE, respectively, in exposed mice (1,000 and 2,000 ppm combined) between genotype groups. Histopathologic scores were compared using a nonparametric method (Steel-Dwass method). The alpha level for statistical significance was set at *p* < 0.05.

## Results

### Liver and body weight

We found significant differences among control (unexposed) mice according to genotype (Table 1). Specifically, the mean body weight of h*PPAR*α mice was 14% less and 8.5% less than m*PPAR*α and *Ppar*α-null mice, respectively, and the mean liver weight of h*PPAR*α mice was 11% less than *Ppar*α*-*null mice; the liver/body weight ratio of *Ppar*α-null mice was 11% higher than in m*PPAR*α mice. TRI at 1,000 and 2,000 ppm significantly increased liver weight in the three mouse lines, and the increases were almost the same: 38% and 49% in m*PPAR*α mice; 20% and 37% in *Ppar*α-null mice; and 28% and 32% in h*PPAR*α mice, respectively. However, the increases were not significantly different between TRI doses within each strain. Liver/body weight ratios were also significantly increased with TRI exposure at 1,000 and 2,000 ppm relative to controls (38% and 43% in m*PPAR*α; 24% and 36% in *Ppar*α-null, and 27% and 39% in h*PPAR*α mice, respectively), with a significant difference between 2,000 and 1,000 ppm exposures in *Ppar*α-null mice.

### Urinary metabolites

We observed no differences in the volume of urine samples collected according to genotype or exposure concentration (data not shown). TCA and TCE levels were below the detection limit in all control mice (Table 1). TCA and TCE were detectable in all TRI-exposed mice but were not significantly different between the two TRI exposures within strains. TCA concentrations were significantly lower and TCE concentrations tended to be higher in exposed *Ppar*α-null mice relative to exposed m*PPAR*α mice. Mean concentrations of total TRI metabolites (TCA plus TCE) in m*PPAR*α, *Ppar*α-null, and h*PPAR*α mice were 79.1, 97.9, and 73.8 mmol, respectively, with no significant differences among the genotypes.

### Biochemical changes

AST and ALT liver injury biomarkers varied < 10% among control mice in each strain (Table 1). Plasma ALT and AST levels were significantly increased in all exposed mice relative to controls (41–74% and 36–79% higher, respectively), and mean levels within each group were higher, although not significantly different, with exposure to 2,000 versus 1,000 ppm TRI.

### TG in plasma and liver

In unexposed mice, we observed significantly higher plasma TG levels in h*PPAR*α versus m*PPAR*α mice (52%), and significantly higher liver TG levels in h*PPAR*α mice versus m*PPAR*α and *Ppar*α*-*null mice (77% and 30%, respectively) and in *Ppar*α*-*null versus m*PPAR*α mice (36%) (Table 1). Relative to unexposed mice, liver TG levels were significantly higher in *Ppar*α-null mice exposed to 2,000 ppm TRI (113%) and in h*PPAR*α mice exposed to 1,000 (58%) and 2,000 ppm (87%) TRI. However, there were no significant differences in mean plasma or liver TG concentrations between 2,000-ppm and 1,000-ppm TRI mice within groups. Hepatic TG levels were significantly correlated with liver/body ratios of all mice used in this study (*r* = 0.54).

### Histopathological analysis

**Figure 1:**
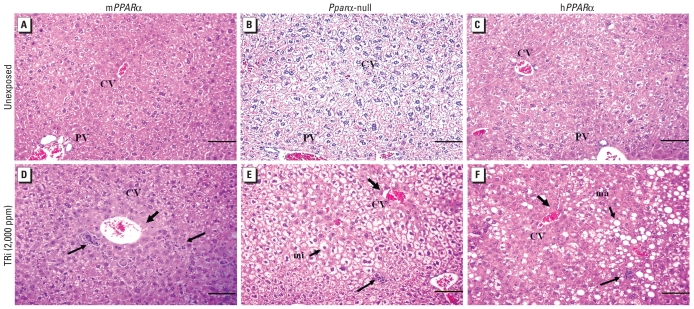
Photomicrographs of representative H&E-stained liver sections from unexposed (*A–C*) and TRI-treated (*D–F*) m*PPAR*α (*A,D*) *Ppar*α-null (*B,E*), and h*PPAR*α (*C,F*) mice. Abbreviations: CV, central vein; PV, portal vein. Treated mice were exposed to 2,000 ppm TRI. Moderate microvesicular steatosis (mi) was observed in exposed *Ppar*α-null mice and macrovesicular steatosis (ma) in exposed h*PPAR*α mice. Thick arrows indicate minimal necrosis around the centrilobular area, and thin arrows denote inflammatory cells. Bars = 100 μm.

We observed neither necrosis nor inflammatory cells in liver sections from unexposed mice [Table 2, Figure 1A–C; see also Supplemental Material, Table 1 (doi:10.1289/ehp.1001928)], which is consistent with lower AST and ALT levels in these mice (Table 1). However, small cytoplasmic vacuoles were present in sections from unexposed *Ppar*α-null and h*PPAR*α mice, similar to the reports by Wolf et al. (2008) and Cheung et al. (2004), respectively, resulting in steatosis scores > 0.

Steatosis was absent in the livers of TRI-exposed and unexposed m*PPAR*α mice, but it was significantly increased in exposed versus unexposed *Ppar*α-null and h*PPAR*α mice; steatosis was significantly higher in *Ppar*α-null mice exposed to 2,000 versus 1,000 ppm TRI [Table 2, Figure 1D–F; see also Supplemental Material, Table 1 (doi:10.1289/ehp.1001928)]. Steatosis scores were significantly correlated with liver TG levels of all mice used in this study (*r* = 0.75). However, macrovesicular steatosis was more common in h*PPAR*α mice than in *Ppar*α-null mice. Necrosis scores were significantly higher in TRI-exposed mice relative to controls in all three genotypes and were significantly higher with 2,000 versus 1,000 ppm TRI exposure in m*PPAR*α and h*PPAR*α mice. Inflammation scores were significantly higher than in controls with exposure to 2,000 ppm TRI in all three groups and significantly higher with 2,000 versus 1,000 ppm TRI in m*PPAR*α mice. Hepatocyte proliferation was significantly increased with 2,000 ppm TRI exposure in m*PPAR*α mice, but there was little evidence of an association with exposure in hPPARα mice and no evidence of proliferation in *Ppar*α-null mice.

### Real-time quantitative PCR

The background expression of several genes differed significantly between strains in control mice (Table 3). Specifically, very long chain acyl-coenzyme A (CoA) dehydrogenase (VLCAD), medium chain acyl-CoA dehydrogenase (MCAD), peroxisomal bifunctional protein (hydratase + 3-hydroxyacyl-CoA dehydrogenase) (PH), peroxisomal thiolase (PT), diacylglicerol acyltransferase 1 (DGAT1), and *p52* mRNA levels were higher in h*PPAR*α mice than in m*PPAR*α and *Ppar*α*-*null mice. mRNA levels for *PPAR*α, proliferation cell nuclear antigen (*PCNA*), *p50*, and tumor necrosis factor alpha (*TNF*α) were higher in h*PPAR*α mice than in m*PPAR*α mice, whereas *PPAR*γ mRNA was lower in h*PPAR*α mice than in *Ppar*α-null mice [Table 3; see also Supplemental Material, Figure 1 (doi:10.1289/ehp.1001928)]. *VLCAD*, *PH*, and *PT* mRNA levels were significantly lower in *Ppar*α-null mice than in m*PPAR*α mice, and *p50*, *p52*, *PPAR*γ, and *TNF*α mRNA levels were higher in *Ppar*α-null mice than in m*PPAR*α mice.

TRI exposure did not increase the expression of human *PPAR*α mRNA in h*PPAR*α mice, but 2,000 ppm TRI significantly increased mouse *PPAR*α mRNA in m*PPAR*α mice (Table 3). *PCNA* mRNA expression and mRNA expression of the PPARα target genes *VLCAD*, *MCAD*, *PH*, and *PT* were increased with TRI exposure relative to controls in m*PPAR*α and h*PPAR*α mice, with more pronounced induction of *PH* and *PT* mRNA in m*PPAR*α mice. However, we observed no significant differences in the expression of these genes between mice exposed to 1,000 versus 2,000 ppm TRI.

Relative to unexposed controls, *DGAT1* and *DGAT2* mRNA significantly increased in h*PPAR*α mice exposed to 2,000 ppm TRI and in *Ppar*α-null mice exposed to 1,000 and 2,000 ppm TRI (Table 3). *PPAR*γ mRNA significantly increased in *Ppar*α-null and h*PPAR*α mice exposed to 1,000 and 2,000 ppm TRI. In contrast, *DGAT1*, *DGAT2*, and *PPAR*γ mRNA levels did not differ with TRI exposure in m*PPAR*α mice.

*NF*κ*B-p65* mRNA expression was significantly increased with TRI exposure in *Ppar*α-null and h*PPAR*α mice but not in m*PPAR*α mice (Table 3). *NF*κ*B*-*p50* mRNA expression was significantly increased with exposure in *Ppar*α-null mice only, but *NF*κ*B*-*p52* and *TNF*α mRNA expression was significantly increased with exposure in all strains. *NF*κ*B*-*p52* mRNA levels were significantly correlated with plasma ALT levels of all mice used in this study (*r* = 0.54).

### Protein expression

Protein expression differed among strains in control mice (Table 3). PPARα levels were 10.4 times higher in unexposed h*PPAR*α mice than in m*PPAR*α mice [see Supplemental Material, Figure 2 (doi:10.1289/ehp.1001928)]. VLCAD, PT, acyl-CoA oxidase (ACOX) A, and ACOX B proteins were significantly higher in unexposed h*PPAR*α mice than in m*PPAR*α and *Ppar*α-null mice, MCAD was lower in unexposed h*PPAR*α and in *Ppar*α-null mice than in m*PPAR*α mice, and NFκB-p65 was lower in h*PPAR*α mice than in *Ppar*α-null mice. VLCAD, MCAD, PH, PT, ACOX A, and ACOX B expression was slightly lower and p65 and p52 expression was slightly higher in unexposed *Ppar*α-null versus m*PPAR*α mice.

Relative to controls, TRI exposure (both doses) significantly increased PPARα target gene proteins VLCAD, PH, PT, ACOX A, and ACOX B in m*PPAR*α and h*PPAR*α mice, but TRI exposure did not induce PPARα protein expression. MCAD protein was significantly increased with TRI exposure in h*PPAR*α mice only. PCNA protein was higher in TRI-exposed mice compared with controls in all strains. NFκB-p52 and TNFα proteins were also increased with TRI exposure in all strains, whereas NFκB-p50 and -p65 proteins were increased only in exposed *Ppar*α-null mice. 4-Hydroxy-2-nonenal protein, a marker of oxidative stress, was increased in *Ppar*α-null mice exposed to 1,000 ppm TRI and in m*PPAR*α and h*PPAR*α mice exposed to 2,000 ppm TRI.

Additionally, we measured hepatic protein expression of CYP2E1 and ALDH2 enzymes, as urine TCA levels were significantly lower in *Ppar*α-null than in m*PPAR*α mice. We did not observe significant differences in the expression of either enzyme among controls (data not shown). TRI exposure did not influence hepatic CYP2E1 expression, but it did decrease ALDH2 expression to a comparable extent in all mouse lines (data not shown).

## Discussion

*Ppar*α-null and h*PPAR*α mice appeared to be more susceptible to TRI toxicity than wild-type m*PPAR*α mice, which suggests that PPARα may help protect mice against TRI-induced hepatotoxicity, especially from lipid accumulation. In particular, we noted increased liver TG levels and steatosis with TRI exposure in *Ppar*α-null and h*PPAR*α mice, but not in m*PPAR*α mice. In contrast, PPARα did not appear to influence plasma AST or ALT concentration or the severity of hepatocyte necrosis and inflammation due to TRI exposure, because those responses were comparable across the three mouse lines. Significant correlations of ALT and AST responses with NFκB-p52 levels underscore the importance of this proinflammatory factor in TRI-induced hepatic damage, as demonstrated previously (Ramdhan et al. 2008), although in *Ppar*α-null mice, NFκB-p65 and -p50 were also elevated after TRI exposure. However, we did not observe substantial differences in ALT, AST, NFκB-p52, or TNFα levels between mice exposed to the two TRI doses (1,000 and 2,000 ppm), regardless of genotype, and we did not observe dose-related differences in the expression of PPARα-target genes in m*PPAR*α or h*PPAR*α mice. These results, along with evidence that hepatic CYP2E1 expression and levels of TRI metabolites (TCA and TCE) were comparable among the three strains, may be consistent with a role of hepatic CYP2E1 in TRI-induced hepatic damage.

Mouse and human PPARα were both activated by TRI exposure, but the functional consequences of activation differed substantially between mouse and human PPARα, with significantly higher *PT* and *PH* mRNA and protein induction by TRI exposure in m*PPAR*α than in h*PPAR*α mice. Similar results were also observed after exposure to the PPARα agonist Wy-14,643 (Cheung et al. 2004). TRI exposure caused significant increases in NFκB-p65 and -p50 expression in *Ppar*α-null mice but had no effect on either factor in m*PPAR*α mice, consistent with complete inhibition of this response by mouse PPARα. Human PPARα appeared to be less effective in inhibiting this pathway, because TRI exposure did induce significant increases in NFκB-p65 expression in h*PPAR*α mice. Additionally, we observed comparable levels of the TRI metabolite and PPARα ligand TCA in the livers of m*PPAR*α and h*PPAR*α mice, which suggests that differences are not related to differences in hepatic exposures between the two strains. Nakamura et al. (2009) also reported that human PPARα activation by microgram-order perfluorooctanoate, a ligand of PPARα, was weaker than mouse PPARα activation in response to the same ligand. Taken together, these results suggest functional differences between human and mouse PPARα. However, such differences were not observed in a cell transfection study (Maloney and Waxman 1999).

There is some evidence that PPARα activation inhibits NFκB-p65 and p50 (Delerive et al. 1999) and protects against liver injury. Our finding that hepatocyte necrosis and inflammatory cell infiltrations were comparable after TRI exposure in all three mouse strains suggests that PPARα might not be important in protecting against the hepatic damage induced by TRI, although we did observe a slight increase in NFκB-p65 and -p50 expression after exposure in *Ppar*α-null mice. On the other hand, TRI exposure was associated with significant increases in NFκB-p52 expression in all three mouse lines, consistent with a previous study (Ramdhan et al. 2008) and with increased expression of the proinflammatory cytokine TNFα. Together, these two factors may then trigger a chain reaction (Elsharkawy and Mann 2007). However, because *NF*κ*B* and *TNF*α mRNA and their proteins were measured using total liver homogenates, we could not distinguish whether the increases were derived from Kupffer cell or hepatocyte responses.

The most intriguing result of the present study may be that, although TRI exposure induced enzyme levels involved in the β-oxidation system to the same degree in both m*PPAR*α and h*PPAR*α mice, the hepatic TG levels in h*PPAR*α mice were increased by TRI exposure in a manner similar to those in *Ppar*α-null mice, but not in m*PPAR*α mice. Histopathological findings also showed that after TRI exposures, lipid accumulation was increased in h*PPAR*α and *Ppar*α-null mice compared with m*PPAR*α mice. TRI exposure significantly increased the expression of *DGAT1* and *DGAT2* genes involved in TG synthesis (Yen et al. 2008) in h*PPAR*α but not m*PPAR*α mice, suggesting that these genes may contribute to TG accumulation in the livers of TRI-exposed h*PPAR*α mice. In *Ppar*α-null mice, hepatic TG and lipid accumulation resulting from a lack of PPARα-mediated fatty acid catabolism may be exacerbated due to elevated DGAT1 and DGAT2 expression in response to TRI exposure. Hepatic TG and lipid accumulations were observed most clearly in *Ppar*α-null mice, followed by h*PPAR*α mice, consistent with an important role of TG-synthesizing enzymes in TRI-induced fat accumulation. TRI-induced effects of DGAT1 and DGAT2 appeared to be negatively related to PPARα activation. However, PPARγ was reported to increase expression of these two genes in rats (Festuccia et al. 2009). TRI induced hepatic *PPAR*γ-mRNA in *Ppar*α-null mice, albeit only slightly in h*PPAR*α mice and not at all in m*PPAR*α mice. Thus, mouse PPARα may inhibit the activation of PPARγ, with the result that PPARγ may not be induced in the livers of m*PPAR*α mice.

Hepatic TG levels were different among the three strains in the absence of TRI exposure, with the highest levels in h*PPAR*α mice and the lowest in m*PPAR*α mice. This suggests that the human PPARα insertion did not restore proper lipid regulation in the liver, but instead resulted in receptor overexpression that exacerbated lipid dysregulation by increasing TG storage and steatosis. Higher constitutive expression of DGAT1 may contribute to these effects, but further experiments are needed to pinpoint the underlying mechanism.

Similar to results in an earlier study (Ramdhan et al. 2008), we observed a marked hepatomegaly in m*PPAR*α mice exposed to TRI that may be due to cellular hypertrophy resulting from increased numbers of peroxisomes (Nakajima et al. 2000). However, we did not observe cell hypertrophy after TRI exposure in *Ppar*α-null and h*PPAR*α mice, which suggests that hepatomegaly (as indicated by increased liver/body weight ratios) may have been caused by lipid accumulation rather than peroxisome proliferation. Taken together, the underlying mechanism for TRI-induced hepatomegaly in *Ppar*α-null and h*PPAR*α mice was different from that in m*PPAR*α mice. Although Laughter et al. (2004) did not observe an increase in liver/body weight ratio after TRI exposure in *Ppar*α-null mice, Nakajima et al. (2000) reported a result similar to ours. Discrepancies in the nature of the hepatomegaly caused by TRI exposure may reflect differences in exposure routes, duration, or number of animals used in different studies. We measured the cell proliferation marker PCNA (Dietrich 1993) to further investigate hepatocyte proliferation in response to TRI exposure and found that *PCNA* mRNA increased with TRI exposure in m*PPAR*α and h*PPAR*α mice but not in *Ppar*α-null mice, although PCNA protein expression increased with exposure in three groups. However, we observed histopathological evidence of hepatocyte proliferation in response to TRI exposure in m*PPAR*α mice only. This phenomenon should be investigated further.

With regard to TRI metabolism, urinary TCA levels in *Ppar*α-null mice were significantly lower than in m*PPAR*α mice after TRI exposure. To clarify the mechanism underlying the lower TCA levels, we measured expression of the ALDH2 protein involved in the metabolism of chloral hydrate to TCA because TRI has been reported to inhibit ALDH expression and activity (Wang et al. 1999); we found that ALDH2 was reduced to the same degree after TRI exposure in all three strains of mice. Crabb et al. (2001) reported that the PPARα agonist Wy-14,643 also reduced the ALDH2 protein levels by 20–30% in both wild-type and *Ppar*α-null mice. Because hepatic ALDH2 protein expression was inhibited by TRI exposure in all three mouse strains, it is an unlikely cause of the decreased urinary levels of TCA in *Ppar*α-null mice. Other ALDH isozymes may be involved, and further experiments are therefore needed.

In the present study, the differences in background levels of gene expression between unexposed m*PPAR*α and h*PPAR*α mice must be duly noted, along with differences in TRI-induced changes in m*PPAR*α mice observed between the present study and past studies (Ramdhan et al. 2008) and discrepancies between mRNA and protein expression for some genes. The replacement of human PPARα in this study model may not have been sufficient to prevent steatosis and other effects observed in *Ppar*α-null mice; differences in responses between m*PPAR*α and h*PPAR*α mice may reflect functional consequences related to the use of an artificial construct of the reinserted gene, without normal control elements, in addition to or instead of true functional differences between human and mouse PPARα. It also appears that the overexpression of human PPARα in this model may lead to greater background toxicity than is present in the *Ppar*α-null mice, as indicated by steatosis. We also found differences in gene expression between unexposed m*PPAR*α and h*PPAR*α mice. Specifically, *VLCAD*, *MCAD*, *PH*, *PT*, *DGAT1*, *PCNA*, *p52*, and *TNF*α mRNA levels were higher in h*PPAR*α mice than in m*PPAR*α mice, whereas PH and TNFα proteins were comparable, suggesting that mRNA and protein levels do not always correspond with each other, as noted by Ito et al. (2007). Differences in effects of TRI exposure observed between the present and our past study (Ramdhan et al. 2008) included less-pronounced induction of PPARα by TRI exposure and more-pronounced increases in PH protein and *VLCAD* mRNA expression and ALT and AST levels in response to TRI exposure in our previous study using *cyp2e1^+/+^* mice. We also note that urinary TCA levels for m*PPAR*α mice after exposure that we incorrectly reported in our previous study (Ramdhan et al. 2008) have been corrected here. Therefore, although all elements measured were not completely the same, the two studies are consistent overall. Finally, our study was limited by the small number of mice in each group, which may have limited our power to identify statistically significant biological effects.

## Conclusions

CYP2E1 appears to be involved in the generation of intermediate metabolites through which TRI induces liver injury in mice. In contrast, PPARα, and accordingly PPARγ, may be important factors in TRI-induced lipid accumulation in the liver. Because we used genetically modified mice with underlying dysregulation and we evaluated very high TRI exposures that proved systemically toxic, our findings may not directly reveal the difference in “human PPARα function” as determined between mice and humans. Still, evidence of TRI toxicity independent of PPARα status provides valuable information regarding the effects of PPARα genetic manipulation.

## Figures and Tables

**Table 1:** Body and liver weights and concentrations of urinary TRI metabolites, transaminase, and plasma and hepatic TG in m*PPAR*α, *Ppar*α-null, and h*PPAR*α mice after TRI exposure.

Mouse genotype	*n*	TRI (ppm)	Weight (g)		Liver/body weight ratio	TRI metabolites		Transaminase		TG	
			Body	Liver		TCE (mg)	TCA (mg)	AST (IU/L)	ALT (IU/L)	Plasma (mg/dL)	Liver (mg/g)
m*PPAR*α	6	0	25.1 ± 0.7	1.17 ± 0.04	4.68 ± 0.17	ND	ND	19.9 ± 1.3	6.1 ± 0.8	70.4 ± 17.7	25.7 ± 5.1
	6	1,000	25.0 ± 2.3	1.62 ± 0.15#	6.47 ± 0.16#	4.8 ± 3.7	0.49 ± 0.39##	30.4 ± 3.6#	8.6 ± 1.8#	66.7 ± 22.8	31.3 ± 4.3
	6	2,000	26.1 ± 1.3	1.74 ± 0.11#	6.67 ± 0.48#	6.2 ± 2.0	0.49 ± 0.14##	32.4 ± 10.1#	9.2 ± 1.0#	86.0 ± 25.0	33.7 ± 8.5
*Ppar*α-null	5	0	23.6 ± 1.5	1.22 ± 0.09	5.19 ± 0.31*	ND	ND	18.4 ± 1.0	6.0 ± 1.4	77.3 ± 12.7	35.0 ± 2.7*
	5	1,000	22.8 ± 0.7	1.47 ± 0.14#	6.43 ± 0.44#	7.5 ± 1.1	0.26 ± 0.09##	26.3 ± 3.1#	9.1 ± 0.9#	116.4 ± 24.7	51.2 ± 6.9
	5	2,000	23.6 ± 1.8	1.67 ± 0.15#	7.05 ± 0.14#,†	6.4 ± 4.1	0.28 ± 0.17##	32.9 ± 6.6#	10.5 ± 1.3#	102.5 ± 32.4	74.7 ± 22.0#
h*PPAR*α	6	0	21.6 ± 0.7*,**	1.09 ± 0.06**	5.04 ± 0.29	ND	ND	21.0 ± 1.8	5.6 ± 1.0	107.0 ± 30.7*	45.6 ± 13.4*,**
	6	1,000	21.9 ± 2.0	1.40 ± 0.16#	6.39 ± 0.22#	5.7 ± 2.4	0.53 ± 0.17	26.6 ± 5.3#	8.1 ± 1.2#	81.1 ± 17.1	72.2 ± 14.4#
	6	2,000	20.9 ± 1.4	1.44 ± 0.25#	6.98 ± 0.87#	5.0 ± 1.9	0.43 ± 0.21	32.0 ± 5.6#	9.0 ± 2.0#	82.3 ± 35.0	85.4 ± 14.0#

* *p* < 0.05 compared with the m*PPAR*α control group.

** *p* < 0.05 compared with the *Ppar*α-null control group.

# *p* < 0.05 compared with the the control group of the same genotype.

## *p* < 0.05 compared with the same treatment between genotypes.

† *p* < 0.05 between the 1,000 and 2,000 ppm TRI doses.

ND, not detected. Values are mean ± SD. TRI 0 ppm is the control.

**Table 2:** Severity of hepatic injury by histological score (mean ± SD) in m*PPAR*α, *Ppar*α-null, and h*PPAR*α mice after TRI exposure.

	m*PPAR*α			*Ppar*α*-*null			h*PPAR*α		
Histological parameter	Control	TRI 1,000	TRI 2,000	Control	TRI 1,000	TRI 2,000	Control	TRI 1,000	TRI 2,000
Steatosis	0.0 ± 0.0	0.0 ± 0.0	0.0 ± 0.0	0.2 ± 0.1*	1.1 ± 0.2#	1.7 ± 0.5#,†	0.4 ± 0.3*	1.5 ± 0.2#	1.8 ± 0.5#
Necrosis	0.0 ± 0.0	0.7 ± 0.2#	1.2 ± 0.3#,†	0.0 ± 0.0	0.7 ± 0.4#	1.0 ± 0.2#	0.0 ± 0.0	0.6 ± 0.2#	1.0 ± 0.1#,†
Inflammation	0.0 ± 0.0	0.1 ± 0.1	1.0 ± 1.0#,†	0.0 ± 0.0	0.1 ± 0.1	0.3 ± 0.1#	0.0 ± 0.0	0.1 ± 0.2	0.4 ± 0.3#
Hepatocyte proliferation	0.0 ± 0.0	0.2 ± 0.4	0.8 ± 0.4#	0.0 ± 0.0	0.0 ± 0.0	0.0 ± 0.0	0.0 ± 0.0	0.2 ± 0.4	0.3 ± 0.5

* *p* < 0.05 compared with the m*PPAR*α control group.

# *p* < 0.05 compared with the control group in the same genotype.

† *p* < 0.05 compared between the 1,000 and 2,000 ppm TRI doses.

**Table 3:** mRNA and protein expression of several genes.

	m*PPAR*α mice			*Ppar*α-null mice				h*PPAR*α mice				
		TRI (ppm)				TRI (ppm)					TRI (ppm)	
	Control	1,000	2,000	Control	A	1,000	2,000	Control	B	C	1,000	2,000
mRNA												
*PPAR*α	0.024 ± 0.008	–	1.5					1.95 ± 0.43	82		–	–
*VLCAD*	0.59 ± 0.07	1.3	1.6	0.29 ± 0.06	0.49	–	–	0.83 ± 0.07	1.4	2.9	1.4	1.3
*MCAD*	0.30 ± 0.04	1.7	1.9	0.29 ± 0.08	–	–	–	0.42 ± 0.15	1.4	1.4	1.5	–
*PH*	0.51 ± 0.16	18.4	17.5	0.45 ± 0.17	0.88	–	–	2.1 ± 1.1	4.1	4.6	5.4	5.6
*PT*	0.40 ± 0.07	4.2	4.4	0.21 ± 0.06	0.51	–	–	1.0 ± 0.5	2.5	4.9	2.3	2.2
*PCNA* (×10^−2^)	3.8 ± 1.5	1.8	1.9	5.9 ± 1.3	1.5	–	–	7.3 ± 2.3	1.9	–	1.5	1.7
*DGAT1*	0.24 ± 0.03	–	–	0.26 ± 0.04	–	1.5	1.5	0.33 ± 0.09	1.4	1.3	–	1.7
*DGAT2*	0.68 ± 0.16	–	–	0.80 ± 0.16	–	1.4	1.5	0.83 ± 0.27	–	–	1.2	1.4
*PPAR*γ (×10^−5^)	5.4 ± 6.5	–	–	15.0 ± 7.5	2.8	2.0	2.8	4.5 ± 3.1	–	0.3	2.5	2.5
NFκB-*p65* (×10^−2^)	5.7 ± 0.8	–	–	6.4 ± 1.8	–	1.6	1.5	7.5 ± 2.2	–	–	1.5	1.4
NFκB-*p50*	10.0 ± 1.1	–	–	14.9 ± 1.2	1.5	1.4	1.3	13.0 ± 2.5	–	–	–	–
NFκB-*p52* (×10^−3^)	2.9 ± 0.3	1.4	1.5	3.4 ± 0.5	1.2	1.6	1.6	4.0 ± 0.9	1.4	1.2	–	1.4
*TNF*α (×10^−5^)	2.0 ± 1.8	2.4	2.5	6.5 ± 2.0	3.3	–	1.8	7.3 ± 2.2	3.7	–	1.7	1.5

Protein												
PPARα	0.58 ± 0.05	–	–					6.1 ± 0.6	10.4		–	–
VLCAD	0.43 ± 0.12	5.3	4.9	0.33 ± 0.04	–	–	–	1.3 ± 0.1	3.0	3.9	1.8	1.8
MCAD	1.7 ± 0.2	–	–	1.2 ± 0.1	0.69	–	–	1.1 ± 0.1	0.64	–	1.3	1.3
PH	0.64 ± 0.13	4.8	4.1	0.45 ± 0.10	0.71	–	–	0.56 ± 0.12	–	–	4.3	4.6
PT	0.76 ± 0.14	3.7	3.7	0.61 ± 0.06	0.81	–	–	1.5 ± 0.1	2.0	2.5	1.7	1.6
ACOX A	1.5 ± 0.2	1.8	1.7	0.94 ± 0.09	0.62	–	–	2.2 ± 0.2	1.5	2.4	1.2	1.3
ACOX B	2.0 ± 0.3	1.5	1.5	1.5 ± 0.1	0.75	–	–	2.6 ± 0.3	1.3	1.8	–	1.2
PCNA	0.50 ± 0.14	2.4	2.3	0.36 ± 0.09	0.72	1.8	1.9	0.39 ± 0.14	–	–	2.3	2.6
NFκB-p65	0.88 ± 0.23	–	–	1.5 ± 0.3	1.8	1.5	1.4	0.96 ± 0.18	–	0.62	–	–
NFκB-p50	0.48 ± 0.14	–	–	0.43 ± 0.07	–	–	1.4	0.44 ± 0.17	–	–	–	–
NFκB-p52	0.42 ± 0.08	1.8	1.9	0.60 ± 0.14	1.4	1.2	1.5	0.49 ± 0.12	–	–	1.5	1.6
TNFα	0.32 ± 0.11	2.2	2.4	0.35 ± 0.13	–	1.7	2.5	0.30 ± 0.09	–	–	2.3	2.5
4–HNE	5.2 ± 0.7	–	1.4	5.5 ± 0.7	–	1.4	–	5.2 ± 0.7	–	–	–	1.3
ALDH2	2.3 ± 0.1	0.69	0.74	2.7 ± 0.2	–	0.66	0.57	2.4 ± 0.2	–	–	0.56	0.64

Abbreviations: – no significant differences were observed; A, *Ppar*α-null mice*/*m*PPAR*α mice; B, h*PPAR*α mice*/*m*PPAR*α mice; C*,* h*PPAR*α mice/*Ppar*α-null mice. Control values represent the mean ± SD for five or six mice. Values for other exposure groups represent the fold change compared with the control of that group.
